# Clinically deployable AI to predict objective response to radiotherapy-intensified immunotherapy in advanced hepatocellular carcinoma

**DOI:** 10.3389/fonc.2026.1794971

**Published:** 2026-04-02

**Authors:** Lei Tang, Shenshun Tang, Shubo Pan, Lu Xu, Jun Jiang, Zonghao Zhao, Zhenhua Zhang, Lei Peng

**Affiliations:** 1 Department of Infectious Diseases, The Second Affiliated Hospital of Anhui Medical University, Hefei, Anhui, China; 2 Department of General Surgery, The Second Affiliated Hospital of Anhui Medical University, Hefei, Anhui, China; 3 Department of Interventional Radiology, The Second Affiliated Hospital of Anhui Medical University, Hefei, Anhui, China; 4 Department of Oncology, The First Affiliated Hospital of Anhui Medical University, Hefei, Anhui, China; 5 Department of Infectious Diseases, The First Affiliated Hospital of University of Science and Technology of China, Hefei, Anhui, China

**Keywords:** artificial intelligence, hepatocellular carcinoma, immunotherapy, multilayer perceptron, radiotherapy

## Abstract

**Background:**

This study aimed to evaluate whether radiotherapy enhances outcomes in advanced hepatocellular carcinoma (HCC) treated with immune–targeted therapy and to develop an interpretable artificial intelligence model for predicting response.

**Methods:**

In this multicenter retrospective study, 238 patients with HCC receiving immune–targeted therapy across three hospitals were included and categorized into an RT group or a no-radiotherapy (No-RT) group according to whether RT was delivered during treatment. Propensity score matching (PSM) was applied to mitigate baseline imbalance. For objective response rate (ORR) prediction, patients were randomly split (7:3) into training and validation cohorts, and eight AI models were developed and evaluated.

**Results:**

ORR was higher in the RT group than in the No-RT group (43.3% vs 28.8%, P = 0.02). RT was associated with longer overall survival (OS) and progression-free survival (PFS) both before and after PSM (all P < 0.05). Responders exhibited markedly improved OS and PFS compared with non-responders (both P < 0.001). Among eight models, the multilayer perceptron (MLP) achieved the best discrimination in the validation cohort (AUC-ROC = 0.71). SHapley Additive exPlanations (SHAP) highlighted age, tumor size, alpha-fetoprotein (AFP), and RT status as the dominant contributors.

**Conclusions:**

In advanced HCC, adding RT to immune–targeted therapy was associated with improved response and survival. An interpretable MLP model may offer a feasible, clinic-friendly approach to ORR prediction and support individualized immunoradiotherapy decisions.

## Introduction

Hepatocellular carcinoma (HCC) is the dominant histologic subtype of primary liver cancer and remains a leading contributor to cancer mortality worldwide (1). Its epidemiology is notably imbalanced, with particularly high incidence in East Asia and parts of Africa (2). Most HCC develops in the context of chronic hepatic injury and cirrhosis, with major drivers including chronic hepatitis B or C infection, alcohol-associated liver disease, and metabolic dysfunction–associated steatotic liver disease (3, 4). Because early disease is frequently clinically silent and surveillance coverage in at-risk populations is imperfect, many patients are diagnosed at unresectable or advanced stages, and long-term survival remains unsatisfactory (5, 6).

Systemic therapy for advanced HCC has rapidly evolved. Immune checkpoint inhibitor–based combinations with anti-angiogenic targeted agents are now widely used in first-line settings (7–9). Even so, response durability and disease control are still limited for a substantial proportion of patients, and resistance—both primary and acquired—remains a central clinical challenge. Radiotherapy (RT), as a locoregional modality, can provide meaningful tumor control in selected patients (including those with vascular invasion or symptomatic lesions) and may reshape the tumor immune microenvironment, providing biological plausibility for combination strategies with immunotherapy (10–12).

In parallel, artificial intelligence (AI) has begun to reframe oncologic prognostication by enabling integrative modeling across heterogeneous clinical information (13–15). Machine-learning algorithms can capture nonlinear dependencies among clinical variables, laboratory indices, and imaging-derived surrogates that may be difficult to represent in conventional models (16, 17). Importantly, explainable AI approaches can make predictions more transparent by quantifying feature contributions, thereby facilitating clinical interpretability and trust (18).

Against this backdrop, we conducted a multicenter retrospective analysis to develop and validate an AI-based model for predicting objective response in HCC treated with immune–targeted therapy with or without RT. We aimed to provide a timely estimate of therapeutic benefit, identify patients more likely to respond to combined treatment, and offer an interpretable tool to support individualized management and risk-adapted strategies in advanced HCC.

## Methods

### Study design and population

This multicenter retrospective study enrolled consecutive patients with advanced hepatocellular carcinoma (HCC) from three hospitals. In total, 238 patients treated with immune–targeted therapy were included. Patients were categorized into the RT group or the No-RT group based on whether RT was administered during the treatment course.

Eligibility criteria were (1): HCC confirmed by imaging and/or histopathology (2); Barcelona Clinic Liver Cancer (BCLC) stage B or C at treatment initiation; and (3) Child–Pugh class A or B. Exclusion criteria were (1): incomplete clinical or follow-up data (2); refractory ascites or hepatic encephalopathy; or (3) concurrent malignancies other than HCC.

The Second Affiliated Hospital of Anhui Medical University approved this study (SL-YW2022-152). Written informed consent had been obtained from patients at the time of initial treatment. The study was conducted in accordance with the Declaration of Helsinki.

### Imaging assessment and response evaluation

Tumor response was evaluated using modified Response Evaluation Criteria in Solid Tumors (mRECIST) and classified as complete response (CR), partial response (PR), stable disease (SD), or progressive disease (PD). Objective response rate (ORR) was defined as CR + PR.

All imaging studies were independently reviewed by two experienced oncologists and one radiologist. Reviewers were blinded to treatment group assignment and clinical outcomes at the time of assessment. Final response classification (CR, PR, SD, or PD) was determined through joint discussion and consensus among the reviewers.

### Treatment outcomes

Overall survival (OS) was defined as the time from treatment initiation to death from any cause; patients alive at last follow-up were censored on that date. Progression-free survival (PFS) was defined as the time from treatment initiation to the first documented progression per mRECIST or death from any cause, whichever occurred first; patients without events were censored at the last tumor assessment.

### AI model development

All eligible patients were randomly split (7:3) into a training cohort and an independent validation cohort. Eight machine-learning algorithms were developed in the training cohort for ORR prediction: decision tree (DT), elastic net (ENET), k-nearest neighbors (KNN), logistic regression (LR), multilayer perceptron (MLP), random forest (RF), support vector machine (SVM), and extreme gradient boosting (XGBoost). Models trained in the training cohort were evaluated in the validation cohort to assess generalizability and predictive performance. Hyperparameter tuning was conducted within the training cohort using five-fold cross-validation. Only patients with complete baseline data were included in model development, and cases with missing values were excluded (complete-case analysis). No feature standardization was applied prior to model training.

### Statistical analysis

Continuous and categorical variables were compared using the Mann–Whitney U test and chi-square test, respectively. To reduce baseline imbalance between the RT and No-RT groups, propensity score matching (PSM) was performed. The following baseline covariates were included in the propensity score model: age, sex, tumor number, tumor size, hepatitis B virus (HBV) infection status, alpha-fetoprotein (AFP), BCLC stage, portal vein tumor thrombus (PVTT), Child–Pugh class, platelet count (PLT), white blood cell count (WBC), lymph node metastasis, and distant metastasis. Patients in the RT and No-RT groups were matched at a 1:1 ratio using nearest-neighbor matching without replacement. A caliper width of 0.1 of the standard deviation of the logit of the propensity score was applied to restrict poor matches. OS and PFS were estimated by the Kaplan–Meier method and compared using the log-rank test. All tests were two-sided, and P < 0.05 was considered statistically significant.

## Results

### Patient baseline characteristics

Before PSM, the RT and No-RT groups were generally comparable, although the RT group had slightly PLT and lower WBC. Key clinical variables—including tumor number, tumor size, BCLC stage, Child–Pugh class, PVTT, lymph node metastasis, and distant metastasis—were similar between groups. After 1:1 PSM, these imbalances were resolved, and all baseline characteristics were well balanced between groups (**Table 1**).

**Table 1:** Baseline characteristics before and after PSM.

Patients		Before PSM			After PSM		
Characteristic	All N=238	No RT N=111	RT N = 127	P	No RT N=99	RT N = 79	P
Age, years, mean (SD)	53.9 (10.1)	53.9 (10.6)	53.9 (9.70)	0.967	54.5 (10.7)	54.7 (9.68)	0.873
Tumor number ≥ 2	163 (68.5)	82(73.9)	81 (63.8)	0.093	73 (73.7)	59(74.7)	0.887
Size, mean (SD)	7.30 (4.08)	7.27 (3.81)	7.32 (4.32)	0.931	7.02 (3.76)	7.49 (4.26)	0.439
Sex				0.09			0.342
Female	38 (16.0)	23 (20.7)	15 (11.8)		16 (16.2)	8 (10.1)	
Male	200 (84.0)	88 (79.3)	112 (88.2)		83 (83.8)	71 (89.9)	
HBV				0.331			0.228
No	75 (31.5)	31 (27.9)	44 (34.6)		29 (29.3)	16 (20.3)	
Yes	163 (68.5)	80 (72.1)	83 (65.4)		70 (70.7)	63 (79.7)	
Child				0.315			0.821
A	185 (77.7)	90 (81.1)	95 (74.8)		79 (79.8)	65 (82.3)	
B	53 (22.3)	21 (18.9)	32 (25.2)		20 (20.2)	14 (17.7)	
BCLC				0.112			0.714
B	55 (23.1)	20 (18.0)	35 (27.6)		18 (18.2)	17 (21.5)	
C	183 (76.9)	91 (82.0)	92 (72.4)		81 (81.8)	62 (78.5)	
PVTT				0.069			0.697
No	84 (35.3)	32 (28.8)	52 (40.9)		30 (30.3)	27 (34.2)	
Yes	154 (64.7)	79 (71.2)	75 (59.1)		69 (69.7)	52 (65.8)	
N				0.999			0.655
No	134 (56.3)	63 (56.8)	71 (55.9)		57 (57.6)	49 (62.0)	
Yes	104 (43.7)	48 (43.2)	56 (44.1)		42 (42.4)	30 (38.0)	
M				0.072			0.933
No	177 (74.4)	76 (68.5)	101 (79.5)		71 (71.7)	58 (73.4)	
Yes	61 (25.6)	35 (31.5)	26 (20.5)		28 (28.3)	21 (26.6)	
PLT, mean (SD)	160 (74.3)	146 (65.4)	172 (79.5)	0.007	148 (65.5)	171 (85.7)	0.051
WBC				0.045			0.579
<4	44 (18.5)	27 (24.3)	17 (13.4)		22 (22.2)	14 (17.7)	
≥4	194 (81.5)	84 (75.7)	110 (86.6)		77 (77.8)	65 (82.3)	
AFP				0.171			0.663
<400	126 (52.9)	53 (47.7)	73 (57.5)		47 (47.5)	41 (51.9)	
≥400	112 (47.1)	58 (52.3)	54 (42.5)		52 (52.5)	38 (48.1)	

### Treatment outcomes

In the RT group, best responses by mRECIST were CR in 6 patients (4.72%), PR in 49 (38.6%), SD in 54 (42.5%), and PD in 18 (14.2%). In the No-RT group, CR occurred in 3 patients (2.70%), PR in 29 (26.1%), SD in 54 (48.6%), and PD in 25 (22.5%). ORR was higher in the RT group than in the No-RT group (43.3% vs 28.8%, P = 0.02).

In the RT group, 43 patients died and 78 experienced disease progression, whereas in the No-RT group, 48 patients died and 83 experienced progression. RT was associated with improved survival. OS favored the RT group both before and after matching (before PSM, not reached vs 20.8 months, P = 0.022; after PSM, 26.3 vs 20.9 months, P = 0.025). Similar trends were observed for PFS (before PSM, 8.7 vs 6.7 months, P = 0.016; after PSM, 8.7 vs 6.8 months, P = 0.017).

**Figure 1:**
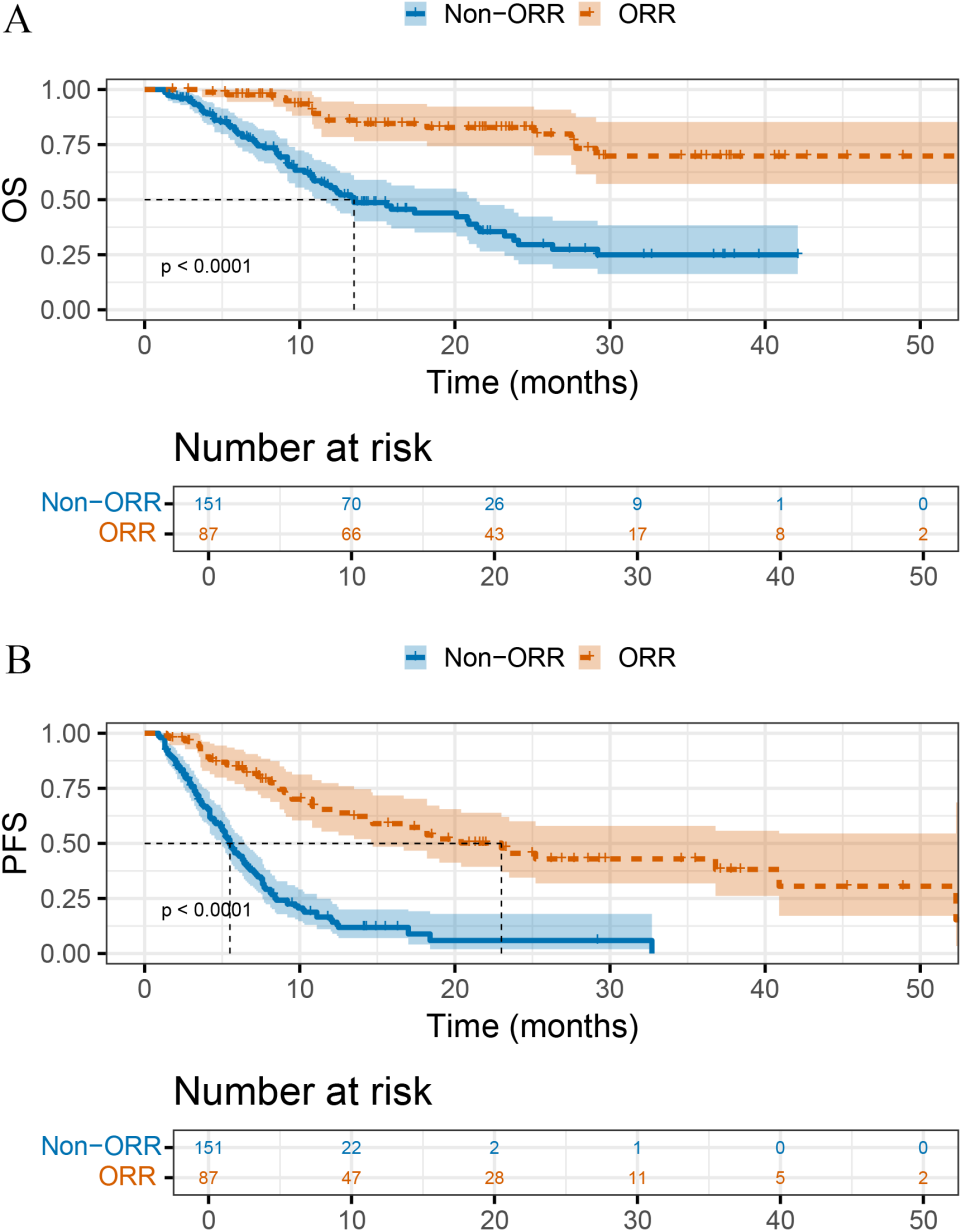
Kaplan–Meier survival curves stratified by objective response. **(A)** Overall survival (OS) and **(B)** progression-free survival (PFS) in patients achieving an objective response (ORR) versus those without objective response (Non-ORR).

Clinical response was strongly linked to long-term outcomes. Responders showed significantly longer OS (not reached vs 13.5 months, P < 0.001, **Figure 1A**) and PFS (23.0 vs 5.5 months, P < 0.001, **Figure 1B**) than non-responders.

### AI-based prediction of ORR: model training and validation

**Figure 2:**
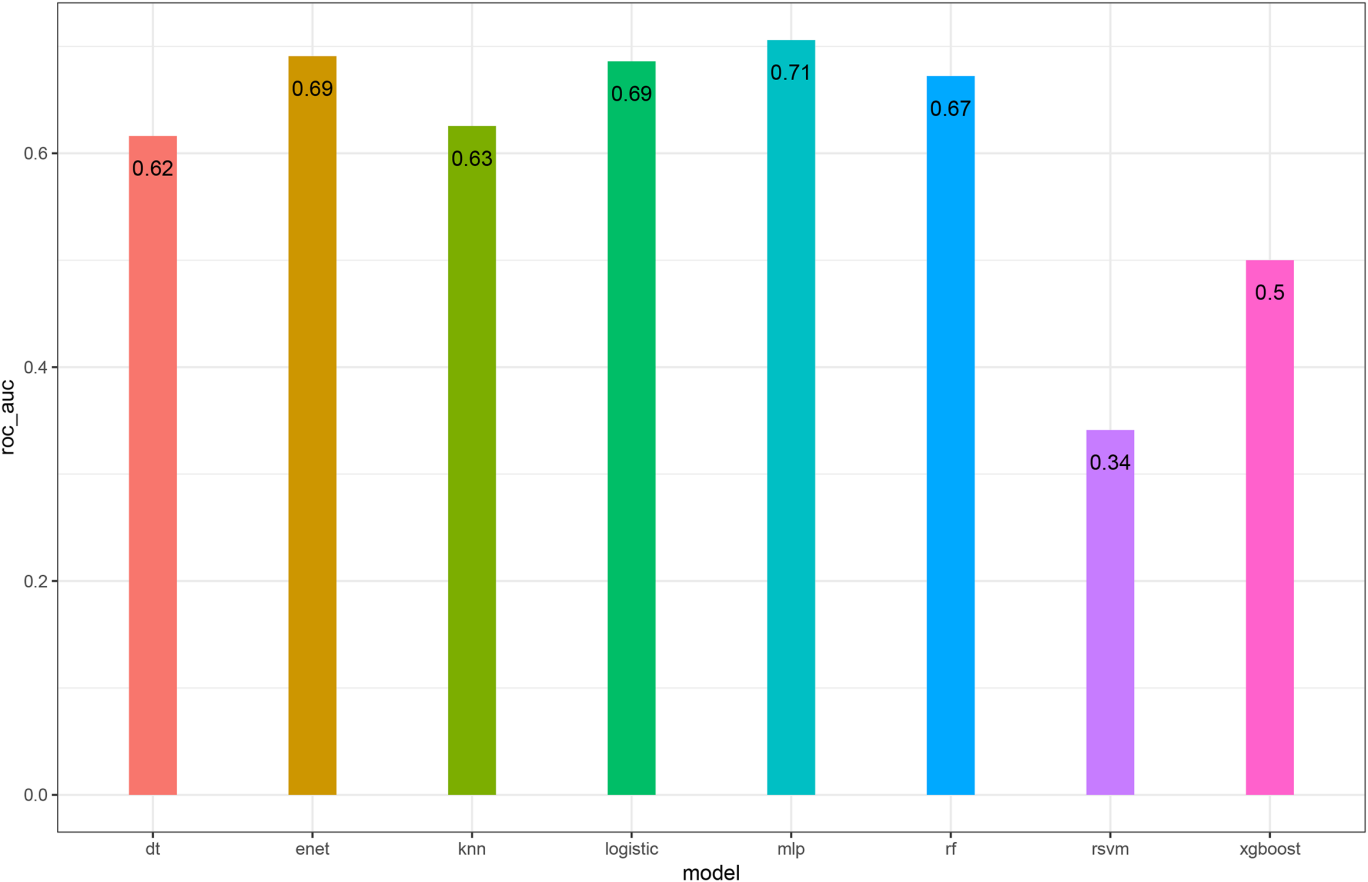
Comparison of model discrimination for ORR prediction in the validation cohort. Bar plot showing the AUC-ROC values of eight machine-learning models (DT, ENET, KNN, logistic regression, MLP, RF, SVM, and XGBoost). The MLP model achieved the highest AUC-ROC (0.71).

**Figure 3:**
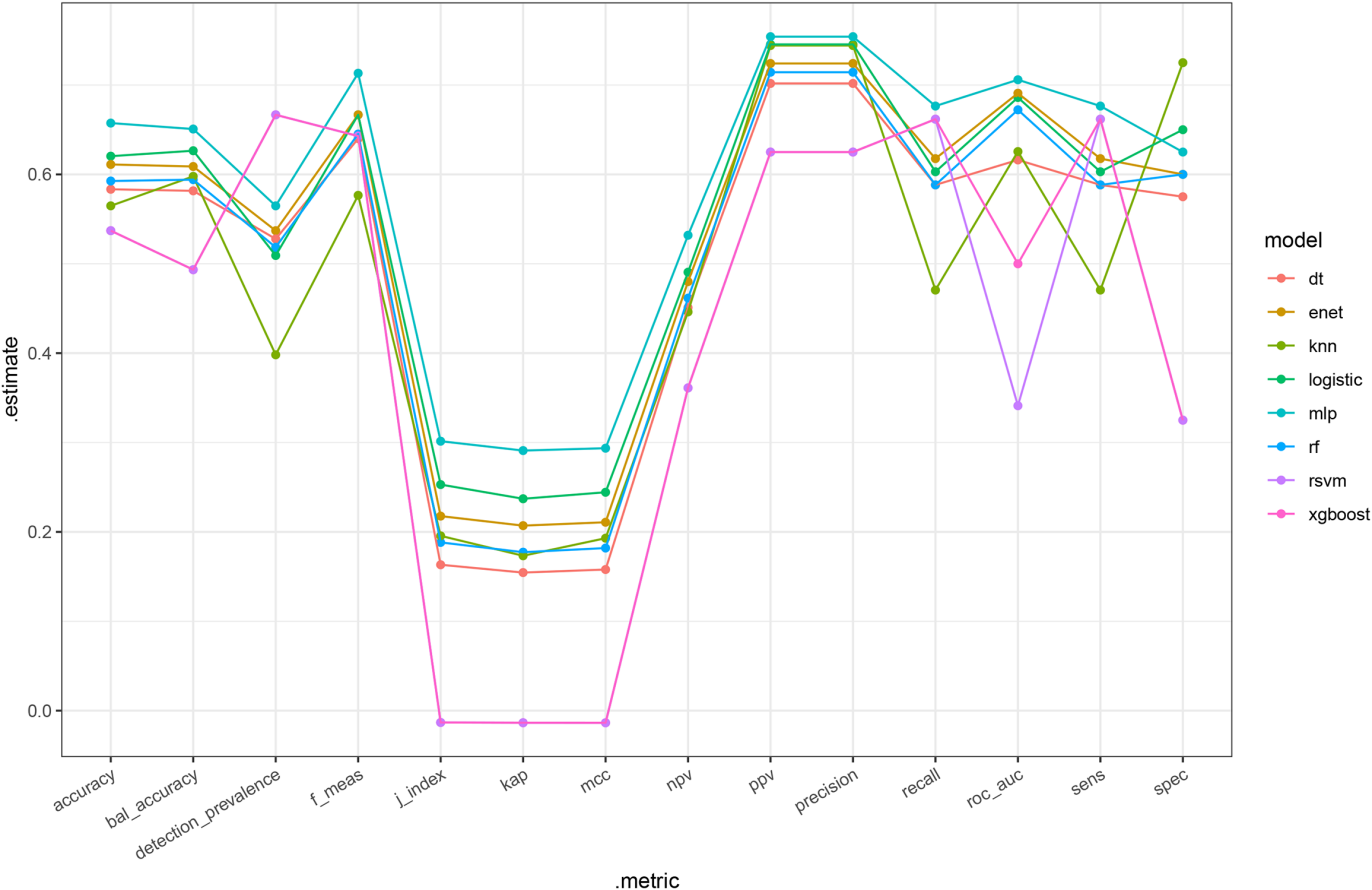
Performance comparison of machine-learning models for ORR prediction in the validation cohort. Line plot summarizing multiple evaluation metrics (accuracy, balanced accuracy, F1 score, J index, kappa, MCC, NPV, PPV, precision, recall, AUC-ROC, sensitivity, and specificity) across eight models.

Patients were randomly split into training and validation sets at a 7:3 ratio. Eight models (DT, ENET, KNN, LR, MLP, RF, SVM, and XGBoost) were trained to predict ORR. In the validation cohort, the MLP model achieved the highest discriminative performance, with an AUC-ROC of 0.71 (**Figure 2**). Across complementary metrics—including accuracy, sensitivity, and specificity—the MLP model also demonstrated favorable performance relative to other algorithms (**Figure 3**).

**Figure 4:**
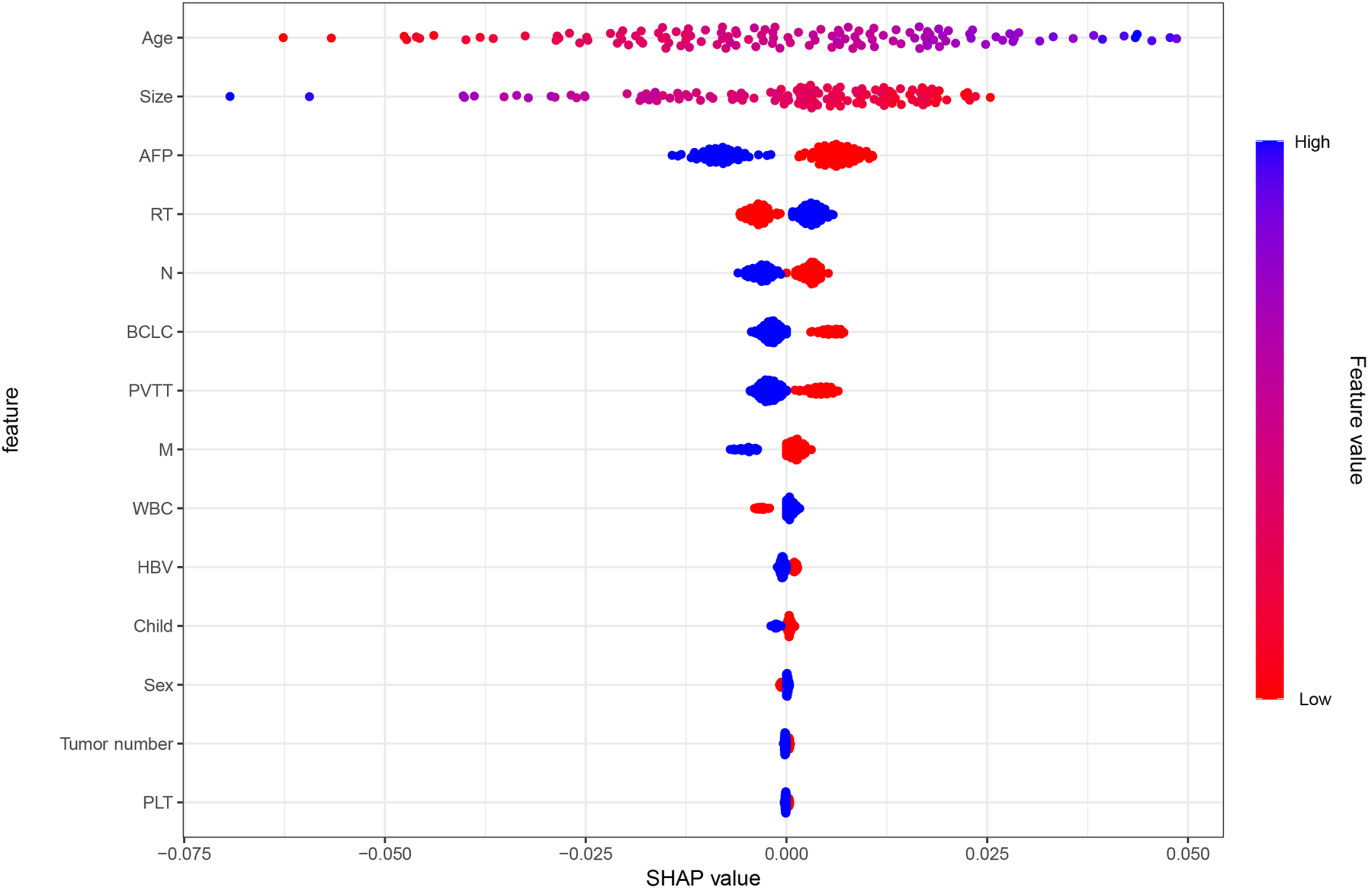
SHAP summary plot for the MLP model in the validation cohort. Features are ranked by overall importance, and each dot represents an individual patient.

Model interpretability was assessed using SHAP in the validation cohort (**Figure 4**). Age, tumor size, and AFP contributed most strongly to prediction, followed by RT, N stage, BCLC stage, and PVTT. Larger tumors and higher AFP generally shifted predictions toward non-response, whereas RT shifted predictions toward response. Feature-importance analysis showed consistent patterns (**Supplementary Figure 1**), with age, tumor size, and AFP as leading contributors and additional signals from N stage and RT status.

## Discussion

In this multicenter retrospective cohort of advanced HCC treated with immune–targeted therapy, RT was associated with higher ORR and longer survival in advanced HCC. The RT group achieved superior ORR compared with the No-RT group, and this advantage translated into longer OS and PFS both before and after propensity score matching. Moreover, responders derived pronounced OS and PFS benefits compared with non-responders, reinforcing objective response as a clinically meaningful correlate of longer-term outcomes in immunotherapy-based regimens.

The clinical signal associated with RT is biologically plausible and likely reflects both direct cytoreduction and immune modulation (19–21). RT can induce immunogenic cell death, increase tumor antigen release and presentation, and reshape the tumor microenvironment toward immune activation, thereby strengthening the rationale for RT–immunotherapy combinations in advanced HCC (22–24). However, real-world benefit is rarely uniform. Differences in tumor burden, liver functional reserve, and metastatic patterns likely produce heterogeneous returns from RT intensification, underscoring the value of tools that can triage patients toward the most promising strategy (25).

To address this need, we developed and validated multiple machine-learning models for ORR prediction, with the MLP model showing the best discrimination in the validation cohort. While the AUC indicates moderate performance, this level of accuracy remains clinically relevant in a complex population where response is shaped by multifactorial tumor biology and competing risks from underlying liver disease. SHAP analysis identified age, tumor size, AFP, and RT status as major contributors to prediction. However, SHAP values represent associations within the observed dataset rather than causal effects. In this observational study, the apparent importance of RT may partly reflect characteristics of patients selected to receive RT (26–28). The alignment between model explanations and clinical reasoning supports the face validity of the approach and may facilitate clinician acceptance.

This study has several practical implications. First, RT may be an effective component of combination strategies for selected advanced HCC patients receiving immune–targeted therapy, even after baseline rebalancing. Second, an AI-based ORR predictor built on readily accessible variables may serve as a feasible decision-support tool to inform patient selection, refine counseling, and guide risk-adapted follow-up.

This study has several practical implications. First, the AI-based ORR predictor may assist clinicians in selecting advanced HCC patients who are more likely to respond to radiotherapy–intensified immune–targeted therapy. Second, it can support shared decision-making by providing individualized response estimates, guide tailored follow-up and surveillance strategies, and help identify patients who may benefit from alternative or additional therapeutic approaches. Because the model uses routinely available features, it may be applied in real-time across centers without specialized assays. Future work may expand this framework to dynamic prediction using longitudinal laboratory trajectories or early imaging changes, potentially improving personalization over the course of therapy (29, 30).

Several limitations should be acknowledged. The retrospective design introduces potential selection bias and unmeasured confounding despite PSM. RT delivery was not necessarily uniform across centers, and factors such as target definition, dose and fractionation, timing relative to systemic therapy, and lesion selection may have influenced outcomes. Response assessment relied on mRECIST, and imaging frequency could vary in routine care, which may affect PFS estimation. The prediction model was internally validated via a random split, and external validation in independent cohorts is required before broader implementation. Claims regarding clinical deployability should be interpreted with caution. Finally, performance may be enhanced by integrating radiomics, treatment-timing variables, or additional biomarkers.

In conclusion, RT added to immune–targeted therapy was associated with higher response and longer survival in advanced HCC, and responders experienced substantial OS and PFS benefit. An interpretable MLP-based model provided a practical approach for ORR prediction and identified clinically coherent predictors dominated by tumor burden and AFP. Prospective studies and external validation are warranted to confirm these findings and refine AI-driven tools for individualized immunoradiotherapy in advanced HCC.

## Data Availability

The original contributions presented in the study are included in the article/**Supplementary Material**. Further inquiries can be directed to the corresponding author.
