# Supplementary material for: Clinically deployable AI to predict objective response to radiotherapy-intensified immunotherapy in advanced hepatocellular carcinoma
Source: Front Oncol. 2026 Apr 2;16:1794971. doi: 10.3389/fonc.2026.1794971 (PMC13083005; doi:10.3389/fonc.2026.1794971)

**Supplementary Fig. 1. Feature importance for the MLP model in the validation cohort.**
Variables are ranked by their contribution to model performance based on the loss function.


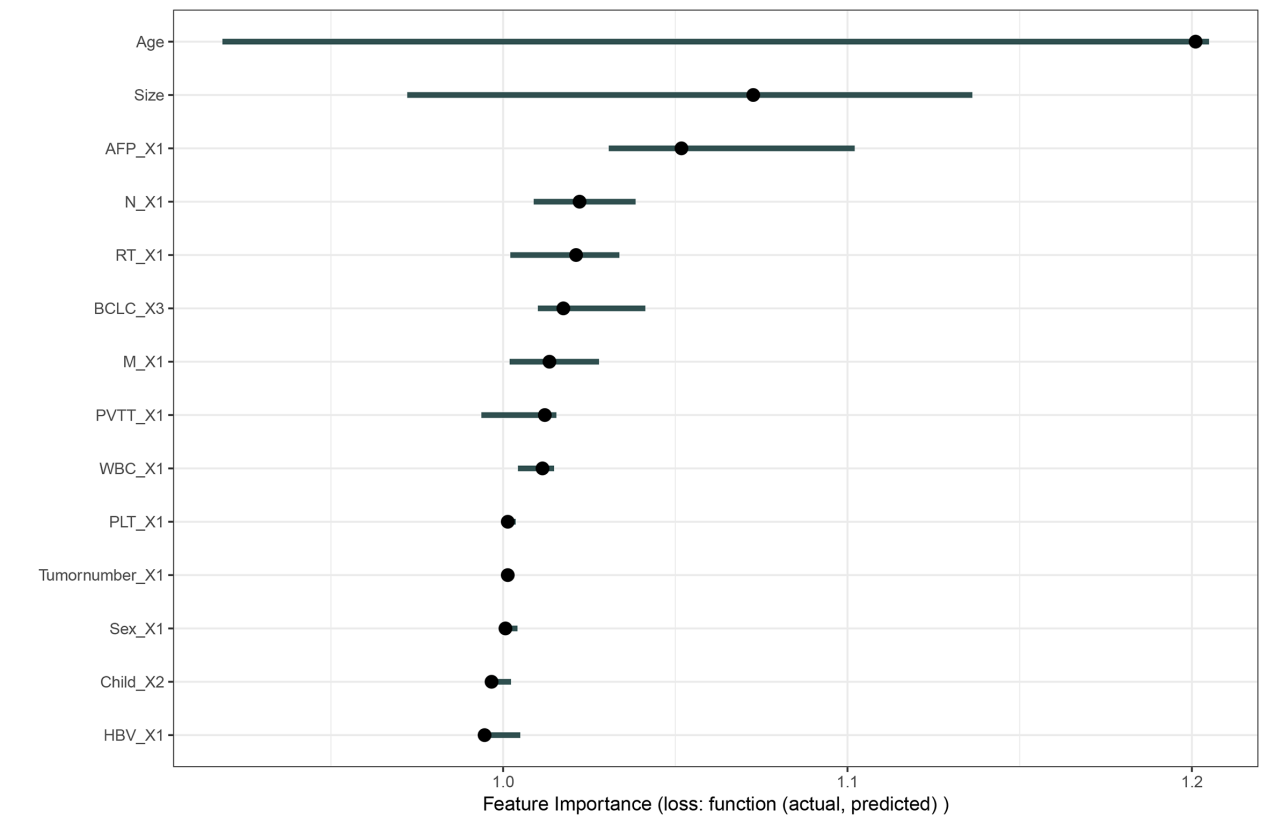

Supplement: Supplementary file 1 [file SupplementaryFile1.docx]
